# Synthesis and crystal structure of bis­(*tert*-butyl isocyanide-κ*C*)[5,10,15,20-tetra­kis­(4-chloro­phen­yl)porphyrinato-κ^4^ *N*]iron(II)

**DOI:** 10.1107/S2056989023008083

**Published:** 2023-09-26

**Authors:** Soumaya Nasri

**Affiliations:** a Department of Chemistry, College of Science, Majmaah University, Al-Majmaah 11952, Saudi Arabia; University of Aberdeen, United Kingdom

**Keywords:** crystal structure, Hirshfeld surface analysis, iron(II) porphyrin complex, *tert* butyl isocyanide

## Abstract

In the title compound, [Fe^II^(C_44_H_24_Cl_4_N_4_)(C_5_H_9_N)_2_] or [Fe^II^(TClPP)(*t*-BuNC)_2_] [where TClPP and *t*-BuNC are 5,10,15,20-tetra­kis­(4-chloro­phen­yl)porphyrinate and *tert-*butyl isocyanide ligands, respectively], the metal ion lies on an inversion center and is octa­hedrally coordinated by the N atoms of the porphyrin ring in the equatorial plane and by carbon atoms of the *trans t*-BuNC ligands in the axial sites. The Fe—N bond length of 2.0074 (14) Å suggests a low-spin complex (*S* = 0). The crystal packing of the title compound is sustained by C—H⋯Cl, C—H⋯N and C^__^H⋯*Cg* (*Cg* = the centroid of a pyrrole ring of the TClPP porphyrinate) inter­actions, leading to a three-dimensional network. The Hirshfeld surface (HS) analysis indicates that 61.4% of the inter­molecular inter­actions are from H⋯H contacts while other contributions are from C⋯H/H⋯C, O⋯H/H⋯O and N⋯H/H⋯N inter­actions, which comprise 21.3%, 13.3% and 3.6% of the HS, respectively.

## Abstract 2

Mol­ecules of the title complex are centrosymmetric and the Fe—N bond lengths to the N atoms of the porphyrin ring indicate that the Fe^II^ atom is in the low-spin state.

## 1. Chemical context

Since the beginning of the 1960s, hexa­coordinated iron(II) metalloporphyrins of type [Fe^II^(Porph)(*L*)_2_], where Porph = porphyrin and *L* is a N-donor neutral axial ligand like pyridine or imidazole, have been widely employed to mimic hemoproteins such as hemoglobin, myoglobin and cytochrome c. These ferrous porphyrin complexes are low-spin 3*d* ^6^ systems (*S* = 0). Such Fe^II^ models with π-acceptor axial ligands like CN^−^, CO and isocyanides (*R*—NC) are also known. Heme-isocyanide derivatives were studied starting from 1951 (St. George & Pauling, 1951 ▸) as a result of their electronic similarity to CO-hemoproteins. Jameson & Ibers (1979 ▸) studied the IR data and the mol­ecular structure of the [Fe^II^(TPP)(*t*-BuNC)_2_] compound. In 1984, the Mössbauer-effect data and the IR of the bis­(isocyanide) iron(II) porphyrin [Fe^II^(TPP)(PhCO-NC)_2_] (PhCO-NC = benzoyl­isocyanide) and the mixed ligand isocyanide-py ligands [Fe^II^(TPP)(PhCO-NC)(py)] were reported (Le Plouzennec *et al.*, 1984 ▸). Subsequently, Salzmann *et al.* (1999 ▸) documented the mol­ecular structure, and undertook ^13^C and ^15^N NMR studies of the [Fe^II^(TPP)(iPrNC)(1-MeIm)] (iPrNC = 2-iso­cyano­propane and 1-MeIm = 1- meth­yl­imidazole) complex. In 2001, spectroscopic investigations of the Fe^II^ and Fe^III^ *n-but­yl* isocyanide complexes P450cam and P450nor were reported (Lee *et al.*, 2001 ▸). In 2017, we reported the spectroscopic and structural characterization of the [Fe^II^(TPBP)(*t*-BuNC)_2_] complex where TPBP is the 5,10,15,20-{tetra­kis-[*para*-(benzo­yloxy)phen­yl]porphyrinate ligand (Nasri *et al.*, 2017 ▸). In order to obtain more insight into the electronic and structural properties of iron(II) bis­(isocyanide) porphyrin complexes, we now report the synthesis, UV/Vis and IR data and the single crystal X-ray structure of the title bis­(*t*-butyl isocyanide)[5,10,15,20-tetra­(*para*-chloro­phen­yl)porphyrinato]iron(II) complex, [Fe^II^(TClPP)(*t*-BuNC)_2_], **I**. 
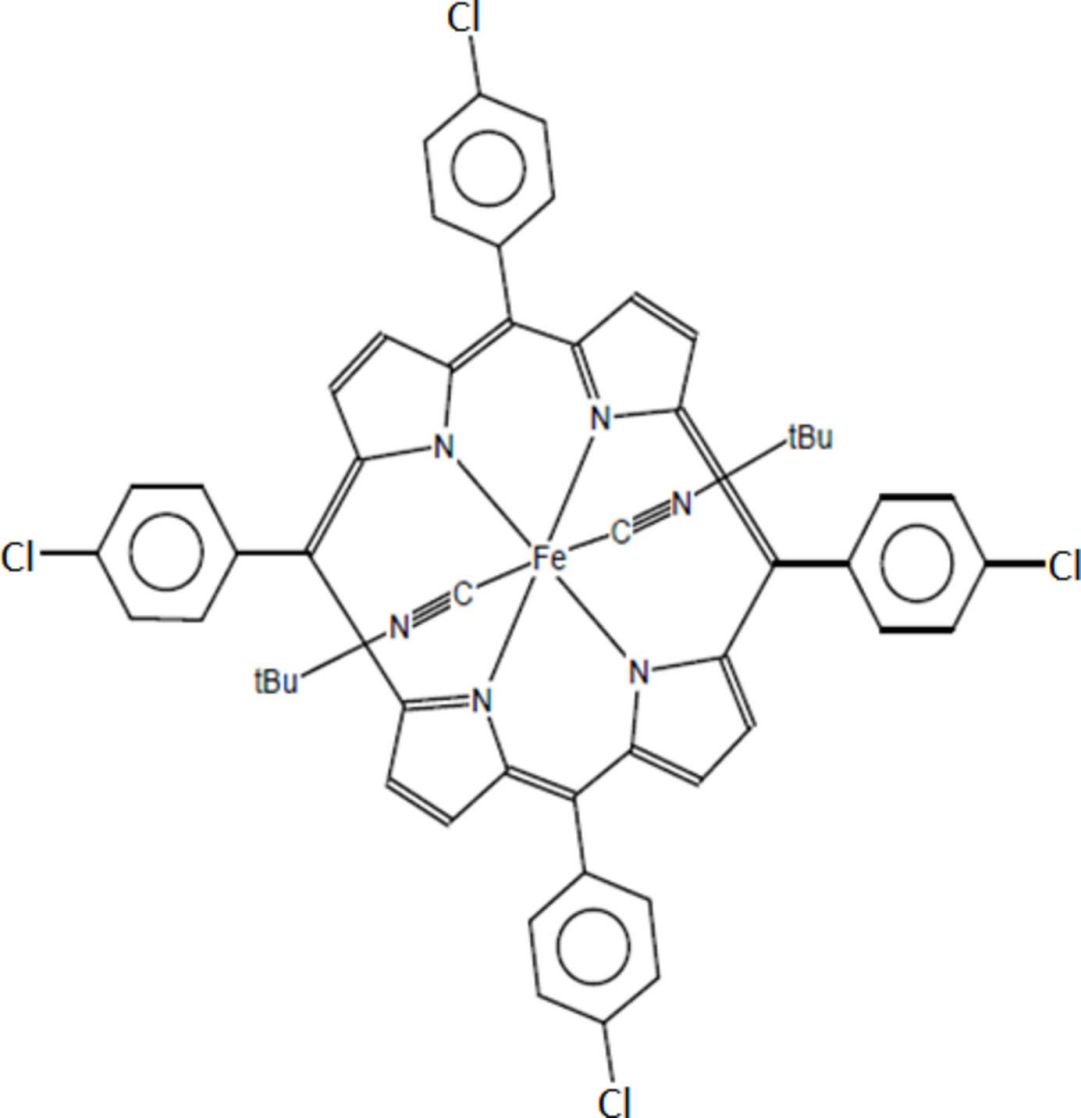


## 2. Structural commentary

**Figure 1:**
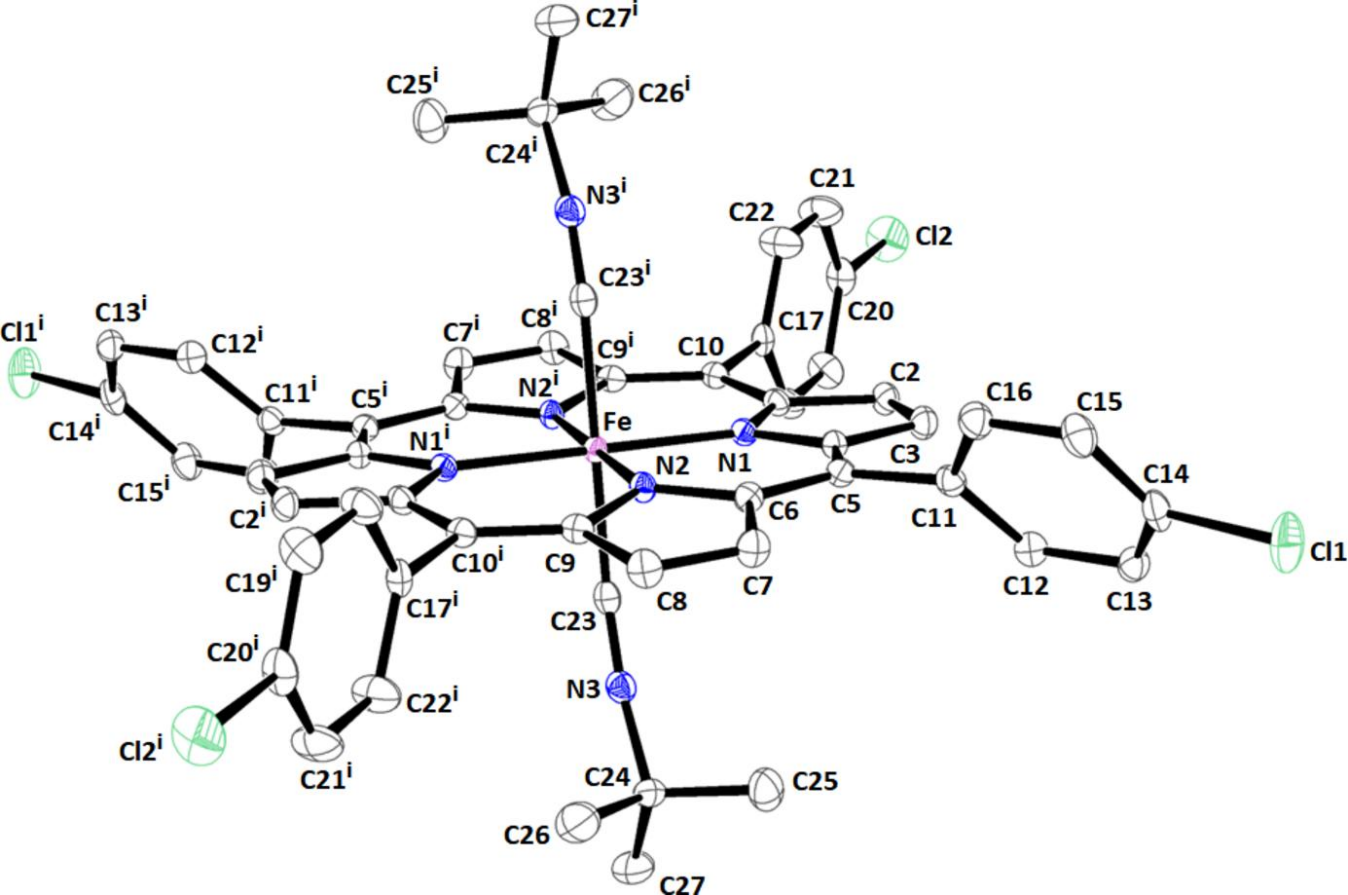
The mol­ecular structure of the title compound with displacement ellipsoids drawn at 40%. The H atoms have been omitted for clarity. Symmetry code: (i) 2 − *x*, −*y*, 1 − *z*.

Complex **I** forms monoclinic crystals (*P*2_1_/*n* space group), wherein the iron(II) atom is positioned on an inversion center. The Fe^II^ center atom exhibits an octa­hedral coordination by four pyrrole N atoms from the porphyrin macrocycle and two *tran*s *t*-BuNC axial ligands. One-half of the [Fe^II^(TClPP)(*t*-BuNC)_2_] mol­ecule comprises the asymmetric unit of complex **I** (Fig. 1 ▸). Scheidt & Reed (1981 ▸) observed a correlation between the average equatorial Fe—N_p_ (p = porphyrin) bond length and the spin state of iron(II) metalloporphyrins. Consequently, in high-spin (*S* = 2) complexes, the Fe—N_p_ distances are the longest, exemplified by the [Fe(TpivPP)(N_3_)]^−^ ion complex (where TpivPP represents α,α,α,α-tetra­kis­(*o*-pivalamido­phen­yl)porphinate, known as the picket-fence porphyrin) where Fe—N = 2.094 (3) Å (Hachem *et al.*, 2009 ▸). In low-spin (*S* = 0) complexes, the average Fe—N_p_ bond length is reduced. For example, in the [Fe^II^(TMPP)(amp)_2_] complex [TMPP is 5,10,15,20-tetra­kis­(4-meth­oxy­phen­yl)porphyrinato and amp is the 4-(2-amino­eth­yl)morpholine], the Fe—N bond length is 1.988 (2) Å (Ben Haj Hassen *et al.*, 2016 ▸). For our ferrous bis­(*t*-BuNC) derivative (**I**), the Fe—N distance of 2.0074 (14) Å strongly suggests that this species corresponds to an iron(II) low-spin (*S* = 0) porphyrin. Notably, this value closely resembles those for the related [Fe^II^(TPP)(*t*-BuNC)_2_] (Jameson & Ibers, 1979 ▸) and [Fe^II^(TPBP)(*t*-BuNC)_2_] (Nasri *et al.*, 2017 ▸) complexes, which are 2.005 (2) and 2.007 (2) Å, respectively.

In complex **I**, the Fe—C distance to the axial ligand measures 1.924 (2) Å, which is very near to those observed in the associated Fe^II^ bis­(*t*-BuNC) metalloporphyrins: [Fe^II^(TPP)(*t*-BuNC)_2_] (where TPP is 5,10,15,20-tetra­phenyl­porphyrinate) and [Fe^II^(TPBP)(*t*-BuNC)_2_] {where TPBP is [4-(benzo­yloxy)phen­yl]porphyrinate} with Fe^II^—C distances of 1.901 (3) and 1.907 (2) Å, respectively (Jameson & Ibers, 1979 ▸ *;* Nasri *et al.*, 2017 ▸). As depicted in Fig. 1 ▸, complex **I** exhibits a non-linear iron(II)–(*t*-BuNC) geometry. Specifically, the Fe—C23—N3 and C23—N3—C24 angles measure 165.75 (15) and 163.66 (17)°, respectively, which closely resemble the corresponding angles observed in [Fe^II^(TPP)(*t*-BuNC)_2_] (Jameson & Ibers, 1979 ▸), which are 170.58 (19) and 167.4 (2)°, respectively.

In the case of the related iron(III) ion complex [Fe^III^(TPP)(*t*-BuCN)_2_]^+^, these angles exhibit significantly higher values, measuring 174.2 and 173.5° for the average Fe—C—N and C—N—C angles, respectively. The deviations from linearity, represented by the angles 14.3/16.3° for complex **I** and 11.0/20.9° for [Fe^II^(TPP)(*t*-BuNC)_2_] (Jameson & Ibers, 1979 ▸), are notably greater than those observed in the iron(III) TPP-bis­(*t*-BuNC) derivative, where the average deviation values are 5.8/6.5° (Walker *et al.*, 1996 ▸). The greater deviation from linearity observed in the *t*-BuCN ligand of ferrous *meso*-metalloporphyrins, compared to the ferric *meso*-porphyrin ion complex [Fe^III^(TPP)(*t*-BuCN)_2_]^+^, is consistent with the dominance of the π-backbonding effect in iron(II) derivatives over iron(III) coordination compounds.

As highlighted in the IR spectroscopy section, the Fe^II^ species exhibit significant π-backbonding, which implies that the C—N bond length in the ferrous *tert-*butyl isocyanide species should be greater than that observed in the ferric *tert*-butyl isocyanide derivatives. Indeed, in the case of **I**, the C—N distance measures 1.159 (2) Å, which is quite similar to the C—N distances observed in the related compound [Fe^II^(TPP)(*t*-BuCN)_2_] (1.152 and 1.162 Å; Jameson & Ibers, 1979 ▸). Comparatively, for the *t*-BuNC–iron(III) ion complexes [Fe^III^(TPP)(*t*-BuNC)_2_]^+^ (Walker *et al.*, 1996 ▸) and [Fe^III^(OEP)(*t*-BuNC)_2_]^+^ (Walker *et al.*, 1996 ▸), the C—N bond length values for the *t*-BuNC ligand are 1.13 (3)/1.12 (2) Å and 1.145 (4)/1.144 (4) Å, respectively.

It is certainly true that the iron(II) derivatives display longer C—N distances. Nevertheless, the difference between the longest C—N bond length of an iron(II)–bis­(*t*-BuNC) derivative and the shortest C—N bond length of an iron(III)–bis­(*t*-BuNC) metalloporphyrin is small (0.042 Å).

## 3. Supra­molecular features

**Figure 2:**
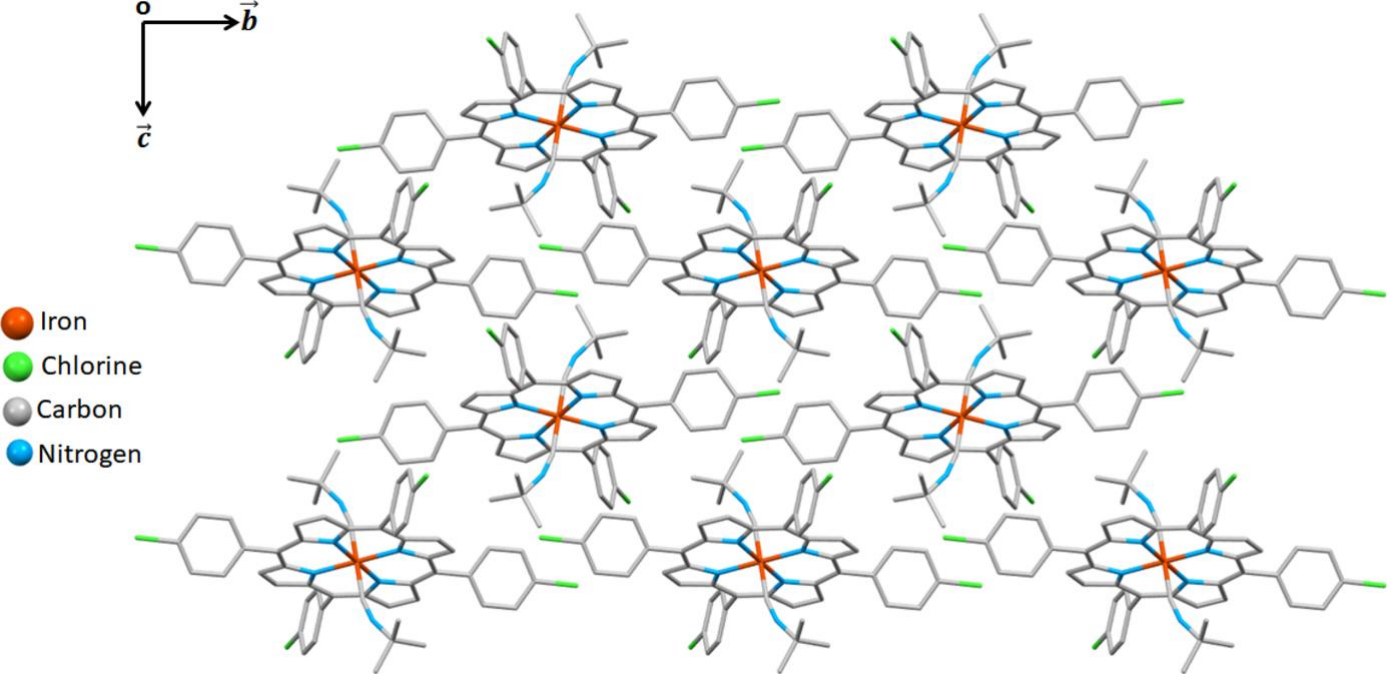
A portion of the crystal packing of the title complex, viewed down [100].

Within the crystal structure of **I** (Figs. 2 ▸ and 3 ▸, Table 1 ▸), the [Fe^II^(TClPP)(*t*-BuNC)_2_] complexes are linked to each other *via* weak non-classical C—H⋯Cl, C—H⋯N hydrogen bonds and C—H⋯*Cg* inter­molecular inter­actions where *Cg* is the centroid of a pyrrole ring. It may be noted that Cl1 acts as acceptor for three different C—H groups.

## 4. Database survey

A search in the Cambridge Structural Database (version 5.43, update of September 2022; Groom *et al.*, 2016 ▸), of iron(II) hexa­coordinated metalloporphyrin complexes type [Fe^II^(Porph)(*L*)_2_] where Porph is a porphyrin and *L* is a N-donor, O-donor, S-donor or C-donor axial ligand gave 25 hits where *L* is a neutral N-donor axial ligand, ten hits for neutral O-donor axial ligands, three S-donor neutral S-donor axial ligands and four neutral C-donor axial ligands. In fact, for the latter type of neutral axial ligands, it is the *tert*-butyl isocyanide corresponding to [Fe^II^(TPP)(*t*-BuNC)_2_] (Jameson & Ibers, 1979 ▸), [Fe^II^(OOEP)(*t*-BuNC)_2_] (OOEP = octa­ethyl­oxophlorinato) (Rath *et al.*, 2004 ▸) and [Fe^II^(TPBP)(*t*-BuNC)_2_] (TPBP = 5,10,15,20-(tetra­kis-[4-(benzo­yloxy)phen­yl]porphyrinate]).

## 5. FT-IR and UV/Vis spectroscopies

The FT–IR spectrum of [Fe^II^(TClPP)(*t*-Bu-NC)_2_] (**I**) (Fig. 4 ▸) was obtained in the 4000–400 cm^−1^ range by a PerkinElmer Spectrum Two FTIR spectrometer. The spectrum exhibits characteristic IR bands of the TClPP porphyrinate. The C—H stretching frequencies of the porphyrin are shown between 3083 and 2923 cm^−1^ while ν(CH) of the methyl groups of the *t*-BuNC axial ligand occurs at 2883 cm^−1^. The strong band at 996 cm^−1^ is attributed to the bonding vibration δ(CCH) of the porphyrin core for which a value around 1000 cm^−1^ is characteristic of a metalled porphyrin while a δ(CCH) value around 960 cm^−1^ is specific of a free base porphyrin.

It has been found that the values of the C≡N stretching frequency for ferric *t*-BuNC metalloporphyrins are displaced by at least 60 cm^−1^ to higher frequency compared to those of ferrous *t*-BuNC porphyrin complexes. Thus for the [Fe^III^(TPP)(*t*-BuNC)_2_]^+^ ion complex (Simonneaux *et al.*, 1989 ▸), the ν(C≡N) frequency value of the *t*-BuNC axial ligand is 2222 cm^−1^ while that of the [Fe^II^(TPP)(*t*-BuNC)_2_] compound (Simonneaux *et al.*, 1989 ▸) is 2129 cm^−1^. Our Fe^II^–TClPP-bis­(*t*-BuNC) species (**I**), exhibits two bands attributed to the ν(C≡N) frequency value of the *t*-BuNC axial ligand with the weak one at 2202 cm^−1^ and the main strong IR band is shown at 2126 cm^−1^. This indicates clearly that complex **I** is iron(II) metalloporphyrin.

The UV/Vis spectrum of complex **I** was obtained in chloro­form using a WinASPECT PLUS scanning spectrophotometer (Fig. 5 ▸). The measurements were conducted in 1.0 cm path length cuvettes containing dry degassed chloro­form solutions, all under an argon atmosphere. The λ_max_ value of the Soret band for complex **I** is 436 nm (Gouterman *et al.*, 1963 ▸), which closely resembles the values observed in the related species [Fe^II^(TPP)(*t*-BuNC)_2_] and [Fe^II^(TBPPP)(*t*-BuNC)_2_], which are 432 nm and 437 nm, respectively (Jameson & Ibers, 1979 ▸; Nasri *et al.*, 2017 ▸). Notably for the bis­(*t*-BuNC) ferric metalloporphyrins, the λ_max_ of the Soret band value is blue shifted compared to those of the ferrous bis­(*t*-BuNC) porphyrin complexes, *e.g*., for [Fe^III^(TPP)(t-BuNC)_2_]^+^ (Simonneaux *et al.*, 1989 ▸), the λ_max_ of the Soret band is 420 nm.

## 6. Hirshfeld surface analysis

**Figure 3:**
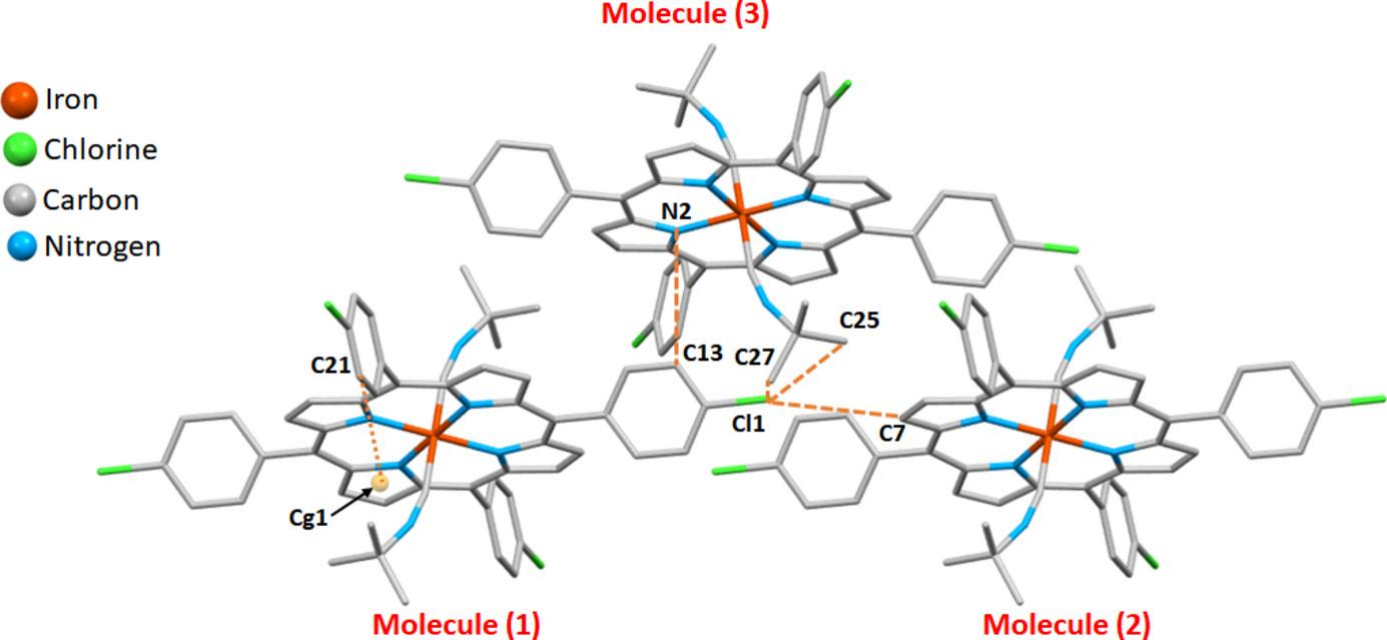
A partial view of the crystal packing of **I** showing the link between the complexes *via* C^__^H⋯Cl and C^__^H⋯N hydrogen bonds and by C—H⋯π inter­actions.

**Figure 4:**
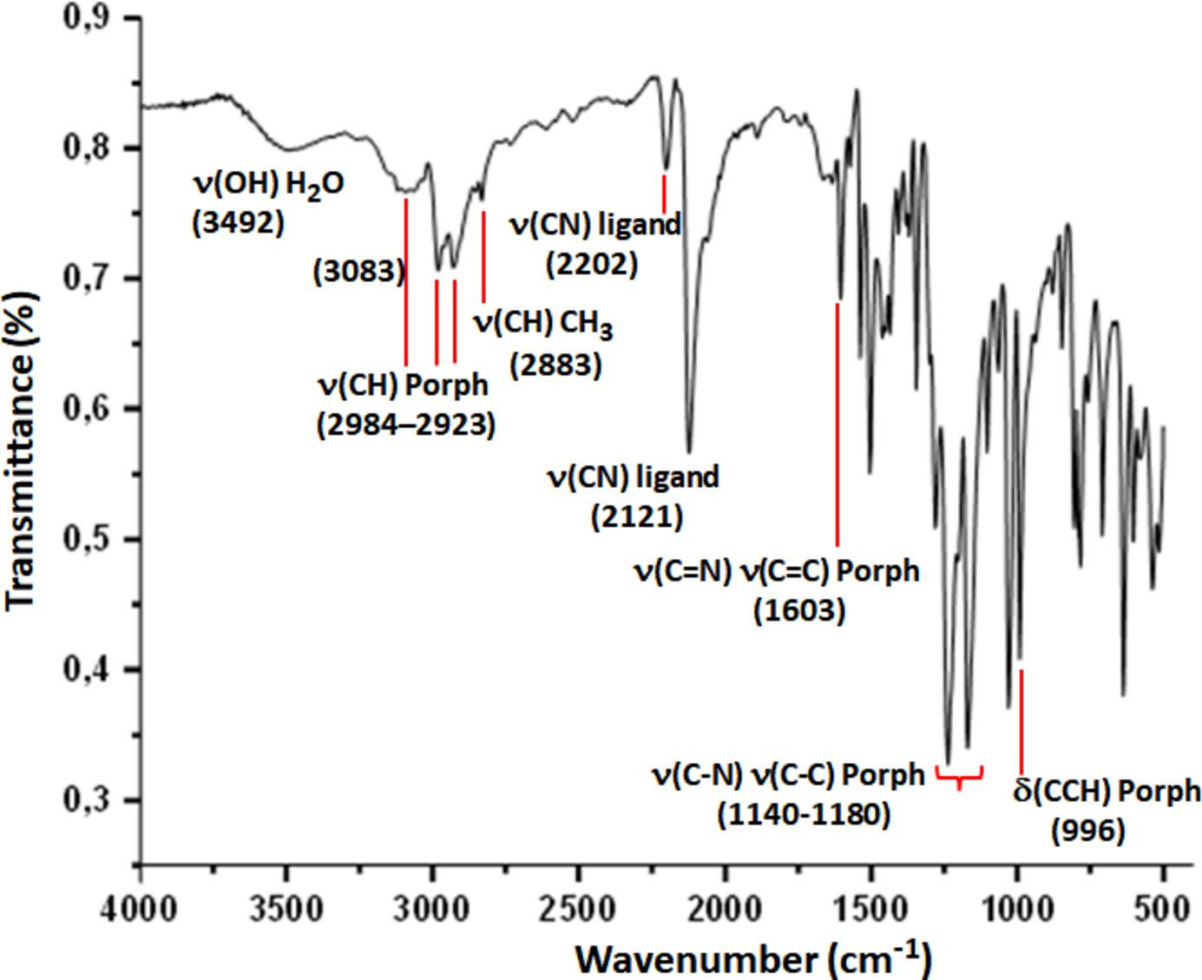
FT–IR spectrum of **I**.

**Figure 5:**
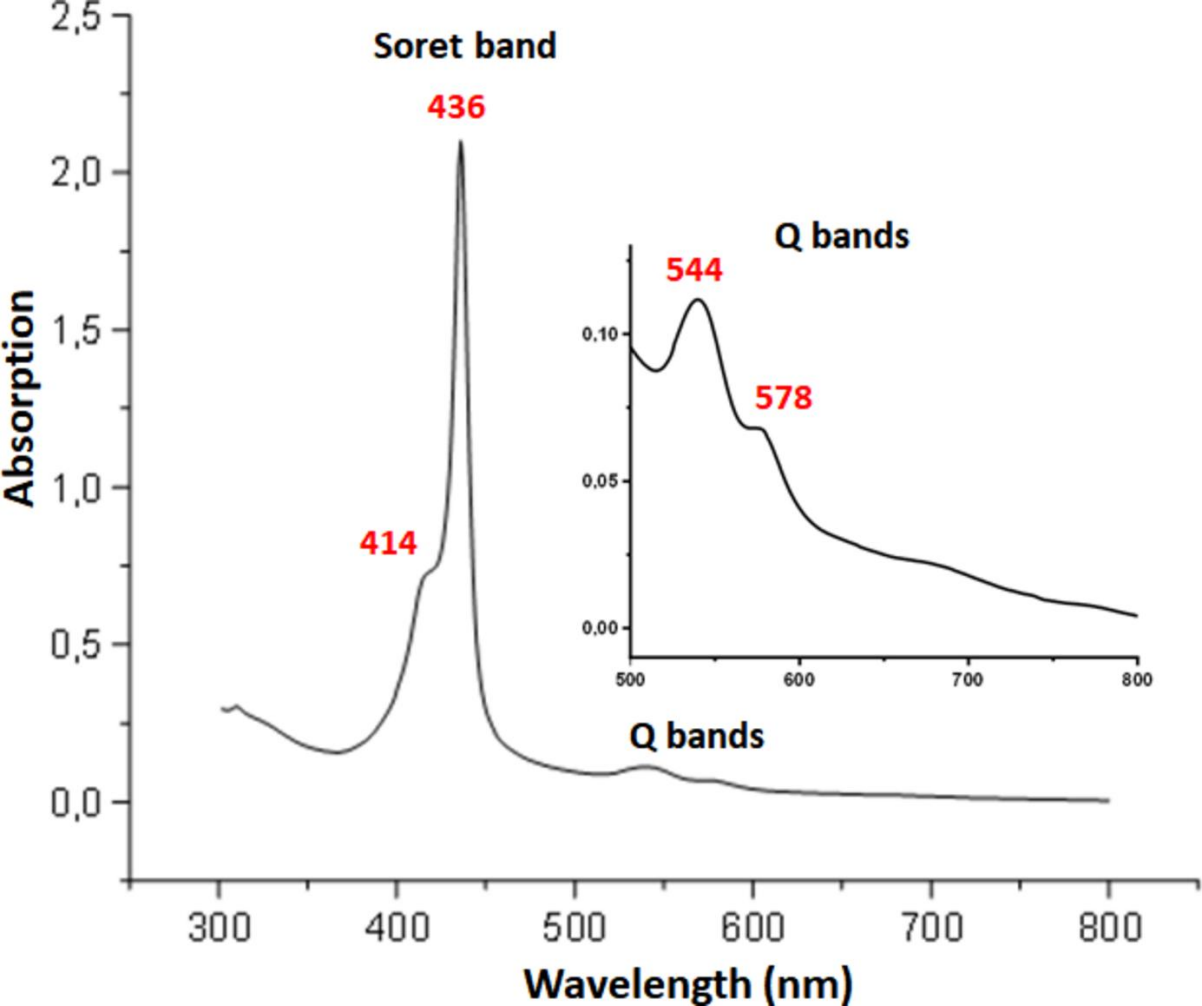
UV/Vis spectrum of **I** recorded in chloro­form.

**Figure 6:**
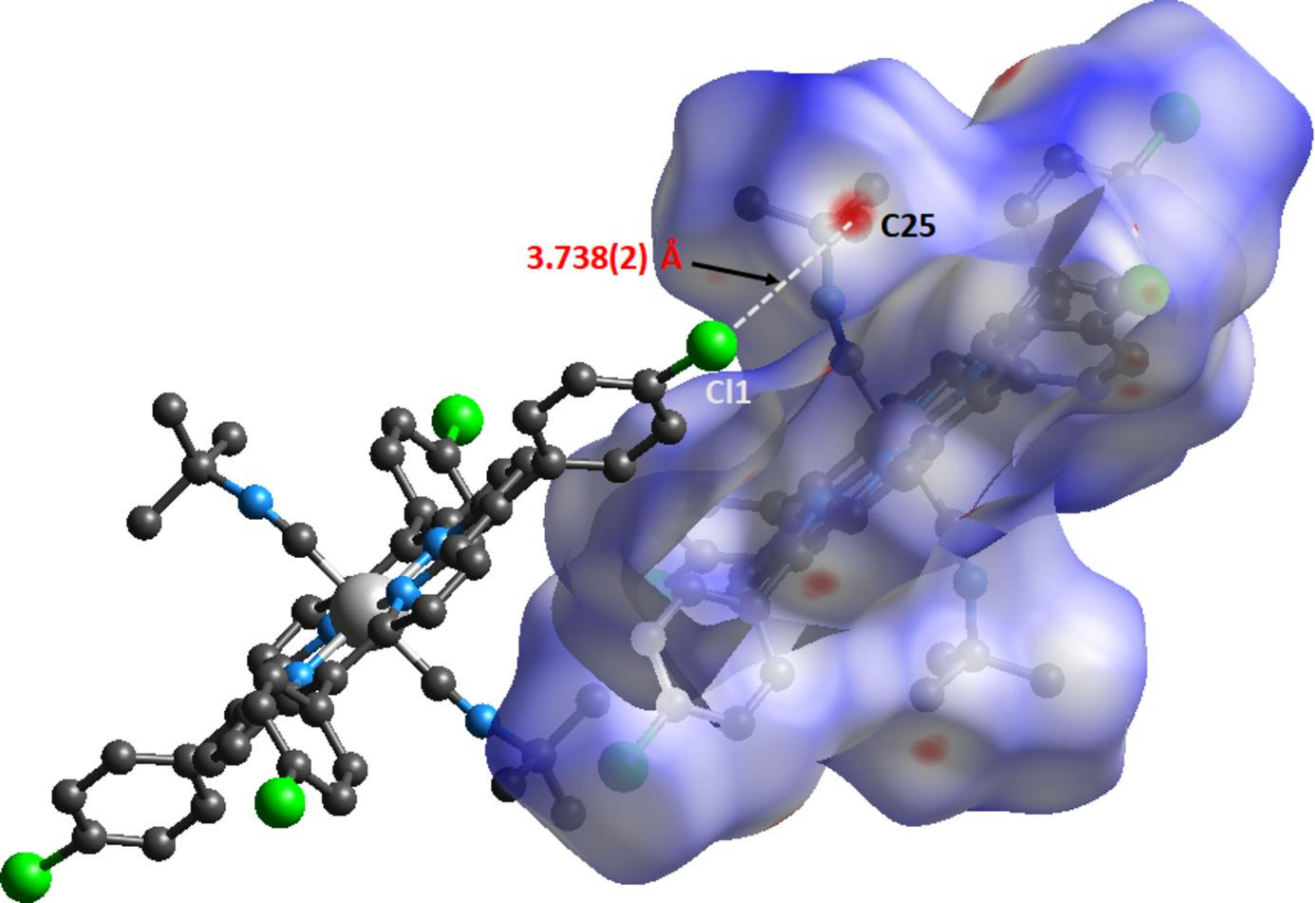
View of the three-dimensional Hirshfeld surface of complex (**I**) plotted over *d*_norm_.

**Figure 7:**
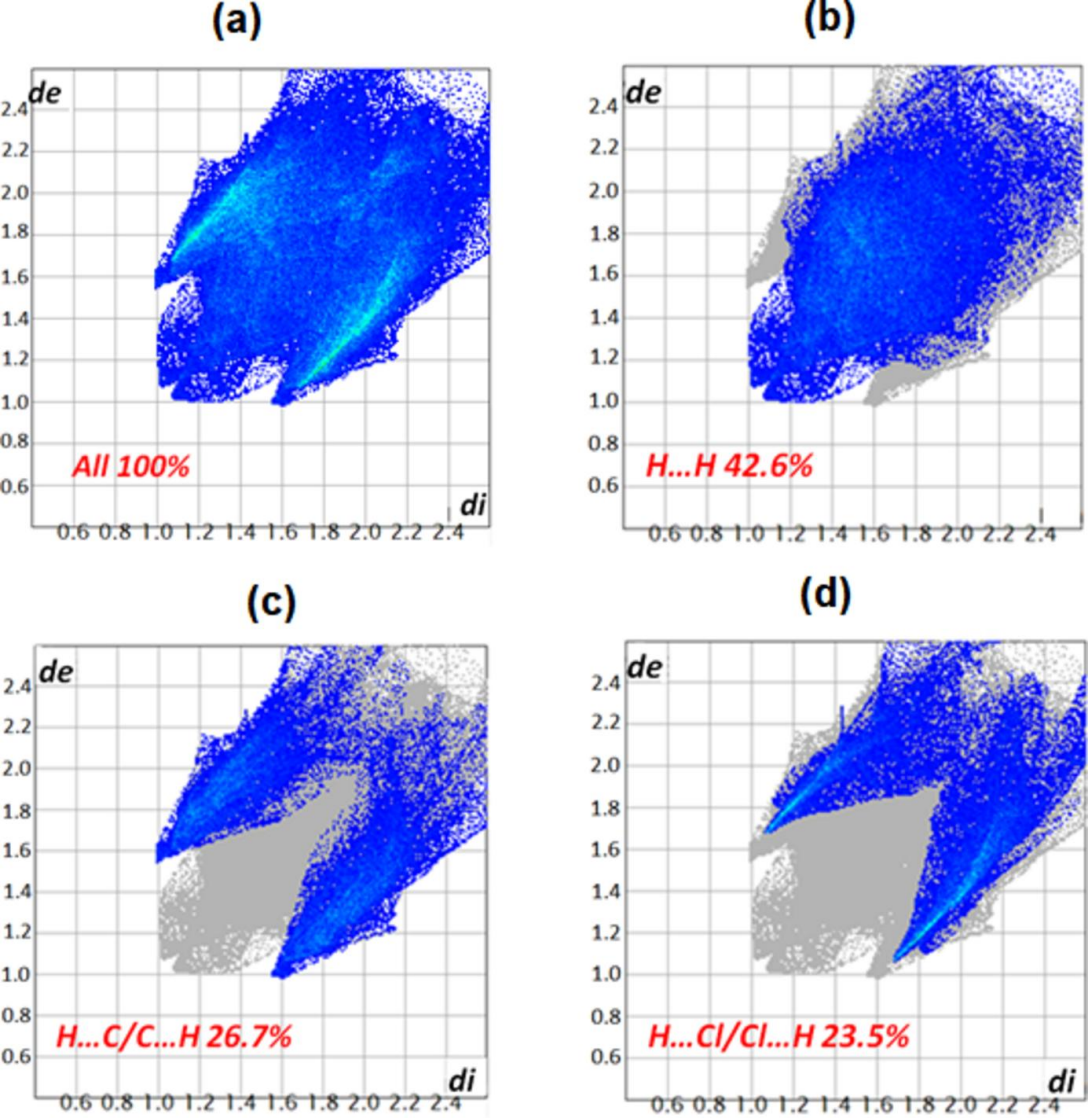
Two-dimensional fingerprint plots of complex **I** showing close contacts of (*a*) all contributions in the crystal and those delineated into (*b*) H⋯H, (*c*) C⋯H/H⋯C and (*d*) Cl⋯H/H⋯Cl inter­actions.

The supra­molecular inter­actions in the title structure have been further investigated and visualized by Hirshfeld surface (HS) analysis performed with *Crystal Explorer 17* (Turner *et al.*, 2017 ▸). The Hirshfeld surface of complex **I** mapped over *d*_norm_ in the range −0.19 to 1.14 a.u. is represented in Fig. 6 ▸. This study confirms that the crystal packing of complex **I** is mainly made by C—H⋯Cl, C—H⋯N and C—H⋯*Cg* inter­molecular inter­actions, as already shown by the *PLATON* program (Spek, 2020 ▸) (Fig. 3 ▸). According to the two-dimensional fingerprint plots of complex **I** shown in Fig. 7 ▸, most important inter­molecular inter­actions are H ⋯H contacts (61.4%). The C⋯H/H⋯C, O⋯H/H⋯O and N⋯H/H⋯N inter­actions comprise 21.3%, 13.3% and 3.6% of the HS, respectively.

## 7. Synthesis and crystallization

The starting materials 5,10,15,20-tetra­(*para*-chloro­phen­yl)porphyrin (H_2_TClPP) and [Fe^III^(TClPP)(SO_3_CF_3_)] were prepared as described in the literature (Adler *et al.*, 1967 ▸; Gismelseed *et al.*, 1990 ▸). To a solution of [Fe^III^(TClPP)(SO_3_CF_3_)] (100 mg, 0.105 mmol) in di­chloro­methane (35 ml) was added an excess of *tert*-butyl isocyanide (*t*-BuNC) (1.2 ml, 10.5 mmol). The reaction mixture was stirred at room temperature for 3 h. Crystals of the title complex were obtained by diffusion of the *n*-hexane and di­chloro­methane solutions. Elemental analysis calculated (%) for C_54_H_42_Cl_4_FeN_6_: C 66.68, H 4.35, N 8.64; found: C 66.81, H 4.41, N 8.78,

## 8. Refinement

Crystal data, data collection and structure refinement details are summarized in Table 2 ▸. All H atoms attached to C atoms were fixed geometrically and treated as riding with C—H = 0.99 Å (methyl­ene) and 0.95 Å (aromatic) with *U*_iso_(H) = 1.2*U*_eq_(C).

## Supplementary Material

Crystal structure: contains datablock(s) I. DOI: 10.1107/S2056989023008083/hb8075sup1.cif

Structure factors: contains datablock(s) I. DOI: 10.1107/S2056989023008083/hb8075Isup2.hkl

CCDC reference: 975663

Additional supporting information:  crystallographic information; 3D view; checkCIF report

## Figures and Tables

**Table 1:** Hydrogen-bond geometry (Å, °) *Cg*1 is the centroid of the N1/C1–C4 pyrrole ring.

*D*—H⋯*A*	*D*—H	H⋯*A*	*D*⋯*A*	*D*—H⋯*A*
C25—H25*C*⋯Cl1^i^	0.98	2.86	3.738 (2)	149
C27—H27*B*⋯Cl1^i^	0.98	2.86	3.733 (2)	149
C7—H7⋯Cl1^ii^	0.95	2.94	3.7953 (18)	151
C13—H13⋯N2^iii^	0.95	2.86	3.685 (2)	146
C21—H21⋯*Cg*1^iv^	0.96	2.69	3.518 (2)	146

Symmetry codes: (i) 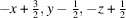 ; (ii) 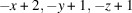 ; (iii) 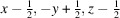 ; (iv) 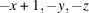 .

**Table 2:** Experimental details

Crystal data	
Chemical formula	[Fe(C_44_H_24_Cl_4_N_4_)(C_5_H_9_N)_2_]
*M*_r_	972.58
Crystal system, space group	Monoclinic, *P*2_1_/*n*
Temperature (K)	115
*a*, *b*, *c* (Å)	10.9679 (5), 16.9240 (7), 13.3536 (5)
β (°)	114.015 (1)
*V* (Å^3^)	2264.15 (16)
*Z*	2
Radiation type	Mo *K*α
μ (mm^−1^)	0.62
Crystal size (mm)	0.28 × 0.21 × 0.18

Data collection	
Diffractometer	Nonius Kappa APEXII
Absorption correction	Numerical (*SADABS*; Krause *et al.*, 2015 ▸)
*T*_min_, *T*_max_	0.903, 0.982
No. of measured, independent and observed [*I* > 2σ(*I*)] reflections	44887, 5201, 4077
*R*_int_	0.052
(sin θ/λ)_max_ (Å^−1^)	0.650

Refinement	
*R*[*F* ^2^ > 2σ(*F* ^2^)], *wR*(*F* ^2^), *S*	0.033, 0.083, 1.05
No. of reflections	5201
No. of parameters	295
H-atom treatment	H-atom parameters constrained
Δρ_max_, Δρ_min_ (e Å^−3^)	0.43, −0.35

Computer programs: *APEX2* (Bruker, 2012 ▸), *SAINT* (Bruker, 2007 ▸), *CrysAlis PRO* (Agilent, 2014 ▸), *SIR2004* (Burla *et al.*, 2005 ▸), *SHELXL* 2013 (Sheldrick, 2015 ▸), *ORTEPIII* (Burnett & Johnson, 1996 ▸), *ORTEP-3 for Windows* (Farrugia, 2012 ▸)and *WinGX* publication routines (Farrugia, 2012 ▸).

## supplementary crystallographic information

### Crystal data

**Table:**

[Fe(C_44_H_24_Cl_4_N_4_)(C_5_H_9_N)_2_]	*F*(000) = 1004
*M**_r_* = 972.58	*D*_x_ = 1.427 Mg m^−^^3^
Monoclinic, *P*2_1_/*n*	Mo *K*α radiation, λ = 0.71073 Å
*a* = 10.9679 (5) Å	Cell parameters from 9956 reflections
*b* = 16.9240 (7) Å	θ = 2.4–27.2°
*c* = 13.3536 (5) Å	µ = 0.62 mm^−^^1^
β = 114.015 (1)°	*T* = 115 K
*V* = 2264.15 (16) Å^3^	Prism, dark violet
*Z* = 2	0.28 × 0.21 × 0.18 mm

### Data collection

**Table:**

Nonius Kappa APEXII diffractometer	5201 independent reflections
Radiation source: X-ray tube	4077 reflections with *I* > 2σ(*I*)
Graphite monochromator	*R*_int_ = 0.052
Detector resolution: 512 x 512 pixels mm^-1^	θ_max_ = 27.5°, θ_min_ = 2.4°
φ and ω scans	*h* = −14→14
Absorption correction: numerical (SADABS; Krause *et al.*, 2015)	*k* = −22→21
*T*_min_ = 0.903, *T*_max_ = 0.982	*l* = −17→15
44887 measured reflections	

### Refinement

**Table:**

Refinement on *F*^2^	0 restraints
Least-squares matrix: full	Hydrogen site location: inferred from neighbouring sites
*R*[*F*^2^ > 2σ(*F*^2^)] = 0.033	H-atom parameters constrained
*wR*(*F*^2^) = 0.083	*w* = 1/[σ^2^(*F*_o_^2^) + (0.0294*P*)^2^ + 1.8163*P*] where *P* = (*F*_o_^2^ + 2*F*_c_^2^)/3
*S* = 1.05	(Δ/σ)_max_ < 0.001
5201 reflections	Δρ_max_ = 0.43 e Å^−^^3^
295 parameters	Δρ_min_ = −0.35 e Å^−^^3^

### Special details

**Table:**

Experimental. SADABS-2012/1 (Bruker,2012) was used for absorption correction. wR2(int) was 0.0545 before and 0.0502 after correction. The Ratio of minimum to maximum transmission is 0.9200. The λ/2 correction factor is 0.0015.
Geometry. All esds (except the esd in the dihedral angle between two l.s. planes) are estimated using the full covariance matrix. The cell esds are taken into account individually in the estimation of esds in distances, angles and torsion angles; correlations between esds in cell parameters are only used when they are defined by crystal symmetry. An approximate (isotropic) treatment of cell esds is used for estimating esds involving l.s. planes.

### Fractional atomic coordinates and isotropic or equivalent isotropic displacement parameters (Å^2^)

**Table:**

	*x*	*y*	*z*	*U*_iso_*/*U*_eq_	
Fe	1.0000	0.0000	0.5000	0.01108 (9)	
C23	1.02702 (17)	0.01547 (10)	0.36771 (14)	0.0145 (4)	
N3	1.05491 (15)	0.03942 (9)	0.29849 (12)	0.0170 (3)	
C24	1.10111 (19)	0.08937 (11)	0.23145 (15)	0.0198 (4)	
C25	1.0239 (2)	0.16655 (11)	0.21248 (19)	0.0276 (5)	
H25A	1.0520	0.2019	0.1677	0.041*	
H25B	1.0418	0.1917	0.2832	0.041*	
H25C	0.9281	0.1558	0.1744	0.041*	
C26	1.2495 (2)	0.10337 (15)	0.29680 (19)	0.0349 (5)	
H26A	1.2841	0.1367	0.2543	0.052*	
H26B	1.2966	0.0526	0.3123	0.052*	
H26C	1.2636	0.1298	0.3660	0.052*	
C27	1.0745 (2)	0.04753 (13)	0.12358 (17)	0.0312 (5)	
H27A	1.1054	0.0808	0.0785	0.047*	
H27B	0.9786	0.0378	0.0845	0.047*	
H27C	1.1226	−0.0029	0.1384	0.047*	
N1	0.82346 (14)	0.05565 (8)	0.43209 (11)	0.0123 (3)	
N2	1.09274 (14)	0.10501 (8)	0.53887 (11)	0.0123 (3)	
C1	0.69880 (17)	0.02084 (10)	0.38631 (14)	0.0137 (3)	
C2	0.59681 (18)	0.08046 (10)	0.34686 (14)	0.0164 (4)	
H2	0.5034	0.0716	0.3103	0.020*	
C3	0.65836 (18)	0.15122 (11)	0.37137 (15)	0.0175 (4)	
H3	0.6166	0.2016	0.3566	0.021*	
C4	0.79981 (17)	0.13574 (10)	0.42436 (14)	0.0137 (3)	
C5	0.89792 (17)	0.19464 (10)	0.46487 (14)	0.0138 (3)	
C6	1.03494 (17)	0.17860 (10)	0.51399 (14)	0.0138 (3)	
C7	1.13680 (18)	0.23832 (10)	0.53796 (15)	0.0176 (4)	
H7	1.1230	0.2937	0.5281	0.021*	
C8	1.25547 (18)	0.20053 (10)	0.57700 (15)	0.0177 (4)	
H8	1.3410	0.2243	0.5995	0.021*	
C9	1.22812 (17)	0.11779 (10)	0.57818 (14)	0.0142 (3)	
C10	0.67285 (17)	−0.05975 (10)	0.38344 (14)	0.0137 (3)	
C11	0.85653 (17)	0.27954 (10)	0.45362 (14)	0.0152 (4)	
C12	0.78007 (18)	0.31308 (10)	0.35164 (15)	0.0172 (4)	
H12	0.7471	0.2803	0.2884	0.021*	
C13	0.75130 (18)	0.39353 (11)	0.34085 (15)	0.0188 (4)	
H13	0.6975	0.4156	0.2714	0.023*	
C14	0.80245 (18)	0.44073 (10)	0.43308 (16)	0.0190 (4)	
C15	0.87414 (19)	0.40905 (11)	0.53618 (16)	0.0217 (4)	
H15	0.9057	0.4420	0.5992	0.026*	
C16	0.89919 (19)	0.32841 (11)	0.54595 (15)	0.0196 (4)	
H16	0.9461	0.3060	0.6166	0.024*	
C17	0.52953 (17)	−0.08653 (10)	0.33800 (14)	0.0148 (4)	
C18	0.46030 (18)	−0.10376 (12)	0.22725 (15)	0.0219 (4)	
H18	0.5034	−0.0973	0.1788	0.026*	
C19	0.32887 (19)	−0.13036 (12)	0.18617 (16)	0.0236 (4)	
H19	0.2825	−0.1420	0.1104	0.028*	
C20	0.26692 (18)	−0.13963 (11)	0.25659 (17)	0.0209 (4)	
C21	0.3315 (2)	−0.12213 (14)	0.36586 (17)	0.0299 (5)	
H21	0.2873	−0.1280	0.4136	0.036*	
C22	0.4631 (2)	−0.09559 (13)	0.40590 (16)	0.0270 (5)	
H22	0.5083	−0.0834	0.4816	0.032*	
Cl2	0.10157 (5)	−0.17304 (3)	0.20542 (5)	0.03464 (14)	
Cl1	0.78308 (5)	0.54293 (3)	0.41772 (5)	0.03197 (14)	

### Atomic displacement parameters (Å^2^)

**Table:**

	*U*^11^	*U*^22^	*U*^33^	*U*^12^	*U*^13^	*U*^23^
Fe	0.00822 (17)	0.01188 (16)	0.01215 (17)	−0.00047 (13)	0.00312 (13)	0.00022 (13)
C23	0.0088 (8)	0.0138 (8)	0.0178 (9)	−0.0010 (6)	0.0024 (7)	−0.0018 (7)
N3	0.0138 (7)	0.0197 (8)	0.0167 (8)	0.0018 (6)	0.0053 (6)	−0.0003 (6)
C24	0.0179 (9)	0.0241 (9)	0.0198 (9)	0.0024 (8)	0.0102 (8)	0.0065 (8)
C25	0.0253 (11)	0.0195 (9)	0.0401 (12)	0.0018 (8)	0.0154 (10)	0.0045 (9)
C26	0.0185 (11)	0.0500 (14)	0.0349 (12)	−0.0002 (10)	0.0095 (9)	0.0147 (11)
C27	0.0425 (13)	0.0337 (12)	0.0243 (11)	0.0067 (10)	0.0207 (10)	0.0053 (9)
N1	0.0107 (7)	0.0130 (7)	0.0132 (7)	−0.0004 (5)	0.0049 (6)	0.0000 (6)
N2	0.0084 (7)	0.0133 (7)	0.0138 (7)	−0.0003 (5)	0.0031 (6)	0.0001 (6)
C1	0.0104 (8)	0.0171 (8)	0.0136 (8)	0.0003 (7)	0.0049 (7)	−0.0003 (7)
C2	0.0101 (8)	0.0192 (9)	0.0175 (9)	0.0013 (7)	0.0032 (7)	0.0019 (7)
C3	0.0145 (9)	0.0178 (9)	0.0194 (9)	0.0024 (7)	0.0060 (7)	0.0025 (7)
C4	0.0123 (8)	0.0155 (8)	0.0132 (8)	0.0025 (7)	0.0051 (7)	0.0016 (7)
C5	0.0151 (9)	0.0137 (8)	0.0132 (8)	0.0009 (7)	0.0062 (7)	0.0000 (7)
C6	0.0149 (9)	0.0128 (8)	0.0136 (8)	−0.0002 (7)	0.0056 (7)	−0.0009 (7)
C7	0.0163 (9)	0.0136 (8)	0.0214 (9)	−0.0022 (7)	0.0060 (8)	−0.0002 (7)
C8	0.0135 (9)	0.0160 (9)	0.0220 (9)	−0.0043 (7)	0.0054 (8)	−0.0023 (7)
C9	0.0124 (8)	0.0171 (8)	0.0128 (8)	−0.0026 (7)	0.0047 (7)	−0.0017 (7)
C10	0.0105 (8)	0.0177 (8)	0.0118 (8)	−0.0012 (7)	0.0036 (7)	−0.0012 (7)
C11	0.0131 (9)	0.0149 (8)	0.0187 (9)	−0.0001 (7)	0.0078 (7)	0.0002 (7)
C12	0.0162 (9)	0.0167 (8)	0.0181 (9)	−0.0008 (7)	0.0062 (7)	−0.0008 (7)
C13	0.0147 (9)	0.0205 (9)	0.0212 (9)	0.0029 (7)	0.0072 (8)	0.0049 (7)
C14	0.0157 (9)	0.0119 (8)	0.0309 (11)	0.0018 (7)	0.0112 (8)	−0.0006 (7)
C15	0.0194 (10)	0.0205 (9)	0.0226 (10)	0.0020 (8)	0.0061 (8)	−0.0055 (8)
C16	0.0191 (9)	0.0205 (9)	0.0176 (9)	0.0029 (7)	0.0058 (8)	−0.0004 (7)
C17	0.0103 (8)	0.0114 (8)	0.0202 (9)	0.0009 (6)	0.0035 (7)	0.0007 (7)
C18	0.0137 (9)	0.0317 (11)	0.0204 (10)	−0.0021 (8)	0.0071 (8)	−0.0027 (8)
C19	0.0150 (9)	0.0300 (10)	0.0215 (10)	−0.0030 (8)	0.0029 (8)	−0.0071 (8)
C20	0.0088 (8)	0.0187 (9)	0.0312 (10)	−0.0037 (7)	0.0041 (8)	0.0001 (8)
C21	0.0173 (10)	0.0489 (13)	0.0257 (11)	−0.0065 (9)	0.0109 (9)	0.0028 (10)
C22	0.0163 (10)	0.0453 (13)	0.0185 (10)	−0.0059 (9)	0.0061 (8)	−0.0032 (9)
Cl2	0.0135 (2)	0.0395 (3)	0.0448 (3)	−0.0117 (2)	0.0056 (2)	−0.0006 (2)
Cl1	0.0304 (3)	0.0139 (2)	0.0455 (3)	0.00429 (19)	0.0091 (2)	0.0003 (2)

### Geometric parameters (Å, º)

**Table:**

Fe—C23	1.9244 (18)	C5—C11	1.496 (2)
Fe—C23^i^	1.9244 (18)	C6—C7	1.443 (2)
Fe—N1^i^	2.0074 (14)	C7—C8	1.350 (3)
Fe—N1	2.0074 (14)	C7—H7	0.9500
Fe—N2^i^	2.0081 (14)	C8—C9	1.434 (2)
Fe—N2	2.0081 (14)	C8—H8	0.9500
C23—N3	1.159 (2)	C9—C10^i^	1.398 (2)
N3—C24	1.464 (2)	C10—C9^i^	1.398 (2)
C24—C26	1.520 (3)	C10—C17	1.506 (2)
C24—C25	1.521 (3)	C11—C12	1.397 (2)
C24—C27	1.523 (3)	C11—C16	1.398 (2)
C25—H25A	0.9800	C12—C13	1.392 (2)
C25—H25B	0.9800	C12—H12	0.9500
C25—H25C	0.9800	C13—C14	1.381 (3)
C26—H26A	0.9800	C13—H13	0.9500
C26—H26B	0.9800	C14—C15	1.386 (3)
C26—H26C	0.9800	C14—Cl1	1.7446 (18)
C27—H27A	0.9800	C15—C16	1.388 (3)
C27—H27B	0.9800	C15—H15	0.9500
C27—H27C	0.9800	C16—H16	0.9500
N1—C4	1.376 (2)	C17—C22	1.383 (3)
N1—C1	1.382 (2)	C17—C18	1.391 (3)
N2—C6	1.376 (2)	C18—C19	1.392 (3)
N2—C9	1.376 (2)	C18—H18	0.9500
C1—C10	1.391 (2)	C19—C20	1.375 (3)
C1—C2	1.438 (2)	C19—H19	0.9500
C2—C3	1.348 (2)	C20—C21	1.370 (3)
C2—H2	0.9500	C20—Cl2	1.7513 (18)
C3—C4	1.443 (2)	C21—C22	1.394 (3)
C3—H3	0.9500	C21—H21	0.9500
C4—C5	1.404 (2)	C22—H22	0.9500
C5—C6	1.400 (2)		
			
C23—Fe—C23^i^	180.0	N1—C4—C3	110.35 (15)
C23—Fe—N1^i^	89.88 (6)	C5—C4—C3	124.23 (16)
C23^i^—Fe—N1^i^	90.12 (6)	C6—C5—C4	123.44 (16)
C23—Fe—N1	90.12 (6)	C6—C5—C11	117.22 (15)
C23^i^—Fe—N1	89.88 (6)	C4—C5—C11	119.32 (15)
N1^i^—Fe—N1	180.00 (5)	N2—C6—C5	126.17 (15)
C23—Fe—N2^i^	97.71 (6)	N2—C6—C7	109.97 (15)
C23^i^—Fe—N2^i^	82.29 (6)	C5—C6—C7	123.69 (16)
N1^i^—Fe—N2^i^	89.75 (6)	C8—C7—C6	106.95 (16)
N1—Fe—N2^i^	90.25 (6)	C8—C7—H7	126.5
C23—Fe—N2	82.29 (6)	C6—C7—H7	126.5
C23^i^—Fe—N2	97.71 (6)	C7—C8—C9	107.09 (16)
N1^i^—Fe—N2	90.25 (6)	C7—C8—H8	126.5
N1—Fe—N2	89.75 (6)	C9—C8—H8	126.5
N2^i^—Fe—N2	180.00 (4)	N2—C9—C10^i^	125.90 (16)
N3—C23—Fe	165.75 (15)	N2—C9—C8	110.34 (15)
C23—N3—C24	163.66 (17)	C10^i^—C9—C8	123.75 (16)
N3—C24—C26	107.17 (15)	C1—C10—C9^i^	124.00 (16)
N3—C24—C25	106.85 (15)	C1—C10—C17	118.33 (15)
C26—C24—C25	110.81 (17)	C9^i^—C10—C17	117.65 (15)
N3—C24—C27	109.23 (16)	C12—C11—C16	118.18 (16)
C26—C24—C27	111.31 (17)	C12—C11—C5	121.71 (16)
C25—C24—C27	111.25 (16)	C16—C11—C5	120.05 (16)
C24—C25—H25A	109.5	C13—C12—C11	121.30 (17)
C24—C25—H25B	109.5	C13—C12—H12	119.4
H25A—C25—H25B	109.5	C11—C12—H12	119.4
C24—C25—H25C	109.5	C14—C13—C12	118.74 (17)
H25A—C25—H25C	109.5	C14—C13—H13	120.6
H25B—C25—H25C	109.5	C12—C13—H13	120.6
C24—C26—H26A	109.5	C13—C14—C15	121.51 (17)
C24—C26—H26B	109.5	C13—C14—Cl1	118.87 (14)
H26A—C26—H26B	109.5	C15—C14—Cl1	119.55 (14)
C24—C26—H26C	109.5	C14—C15—C16	118.98 (17)
H26A—C26—H26C	109.5	C14—C15—H15	120.5
H26B—C26—H26C	109.5	C16—C15—H15	120.5
C24—C27—H27A	109.5	C15—C16—C11	121.10 (17)
C24—C27—H27B	109.5	C15—C16—H16	119.4
H27A—C27—H27B	109.5	C11—C16—H16	119.4
C24—C27—H27C	109.5	C22—C17—C18	117.94 (17)
H27A—C27—H27C	109.5	C22—C17—C10	120.72 (16)
H27B—C27—H27C	109.5	C18—C17—C10	121.34 (16)
C4—N1—C1	105.33 (14)	C17—C18—C19	121.09 (18)
C4—N1—Fe	127.86 (11)	C17—C18—H18	119.5
C1—N1—Fe	126.77 (11)	C19—C18—H18	119.5
C6—N2—C9	105.64 (14)	C20—C19—C18	119.22 (18)
C6—N2—Fe	127.13 (11)	C20—C19—H19	120.4
C9—N2—Fe	126.32 (11)	C18—C19—H19	120.4
N1—C1—C10	125.79 (16)	C21—C20—C19	121.22 (17)
N1—C1—C2	110.18 (15)	C21—C20—Cl2	119.33 (16)
C10—C1—C2	123.93 (16)	C19—C20—Cl2	119.44 (15)
C3—C2—C1	107.24 (16)	C20—C21—C22	118.95 (19)
C3—C2—H2	126.4	C20—C21—H21	120.5
C1—C2—H2	126.4	C22—C21—H21	120.5
C2—C3—C4	106.85 (16)	C17—C22—C21	121.57 (18)
C2—C3—H3	126.6	C17—C22—H22	119.2
C4—C3—H3	126.6	C21—C22—H22	119.2
N1—C4—C5	125.39 (16)		

Symmetry code: (i) −*x*+2, −*y*, −*z*+1.

### Hydrogen-bond geometry (Å, º)

*Cg*1 is the centroid of the N1/C1–C4 pyrrole ring.

**Table:**

*D*—H···*A*	*D*—H	H···*A*	*D*···*A*	*D*—H···*A*
C25—H25*C*···Cl1^ii^	0.98	2.86	3.738 (2)	149
C27—H27*B*···Cl1^ii^	0.98	2.86	3.733 (2)	149
C7—H7···Cl1^iii^	0.95	2.94	3.7953 (18)	151
C13—H13···N2^iv^	0.95	2.86	3.685 (2)	146
C21—H21···*Cg*1^v^	0.96	2.69	3.518 (2)	146

Symmetry codes: (ii) −*x*+3/2, *y*−1/2, −*z*+1/2; (iii) −*x*+2, −*y*+1, −*z*+1; (iv) *x*−1/2, −*y*+1/2, *z*−1/2; (v) −*x*+1, −*y*, −*z*.

## References

[bb1] Adler, A. D., Longo, F. R., Finarelli, J. D., Goldmacher, J., Assour, J. & Korsakoff, L. (1967). *J. Org. Chem.* **32**, 476–476.

[bb2] Agilent (2014). *CrysAlis PRO*. Agilent Technologies, Abingdon, England.

[bb3] Ben Haj Hassen, L., Ezzayani, K., Rousselin, Y., Stern, C., Nasri, H. & Schulz, C. E. (2016). *J. Mol. Struct.* **1110**, 138–142.

[bb4] Bruker (2007). *SAINT*. Bruker AXS Inc., Madison, Wisconsin, USA.

[bb5] Bruker (2012). *APEX2*. Bruker AXS, Inc., Madison, WI, USA.

[bb6] Burla, M. C., Caliandro, R., Camalli, M., Carrozzini, B., Cascarano, G. L., De Caro, L., Giacovazzo, C., Polidori, G. & Spagna, R. (2005). *J. Appl. Cryst.* **38**, 381–388.

[bb7] Burnett, M. N. & Johnson, C. K. (1996). Report ORNL-6895, Oak Ridge National Laboratory, Tennessee, USA.

[bb8] Farrugia, L. J. (2012). *J. Appl. Cryst.* **45**, 849–854.

[bb9] Gismelseed, A., Bominaar, E. L., Bill, E., Trautwein, A. X., Winkler, H., Nasri, H., Doppelt, P., Mandon, D., Fischer, J. & Weiss, R. (1990). *Inorg. Chem.* **29**, 2741–2749.

[bb10] Gouterman, M., Wagnière, G. H. & Snyder, L. C. (1963). *J. Mol. Spectrosc.* **11**, 108–127.

[bb11] Groom, C. R., Bruno, I. J., Lightfoot, M. P. & Ward, S. C. (2016). *Acta Cryst.* B**72**, 171–179. 10.1107/S2052520616003954 PMC4822653 27048719

[bb12] Hachem, I., Belkhiria, M. S., Giorgi, M., Schulz, C. E. & Nasri, H. (2009). *Polyhedron*, **28**, 954–958.

[bb13] Jameson, G. B. & Ibers, J. A. (1979). *Inorg. Chem.* **18**, 1200–1208.

[bb14] Krause, L., Herbst-Irmer, R., Sheldrick, G. M. & Stalke, D. (2015). *J. Appl. Cryst.* **48**, 3–10. 10.1107/S1600576714022985 PMC4453166 26089746

[bb15] Lee, D.-S., Park, S.-Y., Yamane, K., Obayashi, E., Hori, H. & Shiro, Y. (2001). *Biochemistry*, **40**, 2669–2677. 10.1021/bi002225s 11258878

[bb16] Le Plouzennec, M., Bondon, A. & Simonneaux, G. (1984). *Inorg. Chem.* **23**, 4398–4399.

[bb17] Nasri, S., Brahmi, J., Turowska-Tyrk, I., Schulz, C. E. & Nasri, H. (2017). *J. Organomet. Chem.* **846**, 176–184.

[bb18] Rath, S. P., Olmstead, M. M. & Balch, A. L. (2004). *Inorg. Chem.* **43**, 7648–7655. 10.1021/ic0491433 15554629

[bb19] Salzmann, R., McMahon, M. T., Godbout, N., Sanders, L. K., Wojdelski, M. & Oldfield, E. (1999). *J. Am. Chem. Soc.* **121**, 3818–3828.

[bb20] Scheidt, W. R. & Reed, C. A. (1981). *Chem. Rev.* **81**, 543–555.

[bb21] Sheldrick, G. M. (2015). *Acta Cryst.* C**71**, 3–8.

[bb22] Simonneaux, G., Hindre, F. & Le Plouzennec, M. (1989). *Inorg. Chem.* **28**, 823–825.

[bb23] Spek, A. L. (2020). *Acta Cryst.* E**76**, 1–11. 10.1107/S2056989019016244 PMC6944088 31921444

[bb24] St. George, R. C. C. & Pauling, L. (1951). *Science*, **114**, 629–634. 10.1126/science.114.2972.629 14913125

[bb25] Turner, M. J., McKinnon, J. J., Wolff, S. K., Grimwood, D. J., Spackman, P. R., Jayatilaka, D. & Spackman, M. A. (2017). *Crystal Explorer 17*. The University of Western Australia.

[bb26] Walker, F. A., Nasri, H., Turowska-Tyrk, I., Mohanrao, K., Watson, C. T., Shokhirev, N. V., Debrunner, P. G. & Scheidt, W. R. (1996). *J. Am. Chem. Soc.* **118**, 12109–12118.

